# Analysis of population genetic structure from Bucaramanga (Colombia) based on gene polymorphisms associated with the regulation of blood pressure

**Published:** 2012-06-30

**Authors:** Francisco Javier León, Fernando Rondón, Clara Inés Vargas, Myriam Oróstegui, Leonelo Bautista, Norma Cecilia Serrano, María c Páez, Adriana Castillo

**Affiliations:** a School of Medicine, Universidad Industrial de Santander; b School of Biology, Universidad Industrial de Santander; c Genetic Laboratory, Universidad Industrial de Santander; d Epidemiological Observatory for Cardiovascular Disease, Universidad Industrial de Santander; e School of Medicine and Public Health, University of Wisconsin,; f Biomedical Research Center, Universidad Autónoma de Bucaramanga

**Keywords:** Polymorphisms, blood pressure, haplotype GCCTG4b, GRK4 gene, eNOS gene

## Abstract

**Introduction:**

In spite of nearly 40% of variability in blood pressure being explained by genetic factors, the identification of genes associated with essential high blood pressure is difficult to determine in populations where individuals have different genetic backgrounds. In these circumstances it is necessary to determinate whether the population is sub-structured because this can bias studies associated with this disease.

**Objective:**

To determine the genetic structure of the population in Bucaramanga from genetic polymorphisms associated with the regulation of blood pressure: 448G>T, 679C>T y 1711C>T from the gene kinase 4 of the dopaminergic receptor linked to the protein G and Glu298Asp, -786T>C and the VNTR of the intron 4 of the gene of endothelial nitric oxide.

**Methods:**

A sample of 552 unrelated individuals was studied through analysis of restriction fragment length polymorphism. The allelic, haplotypic and genotypic frequencies were calculated, the Hardy-Weinberg equilibrium was determined and a molecular analysis of variance was performed to determine the genetic structure.

**Results:**

Thirty-eight (38) Haplotypes were identified with GCCTG4b being the most frequent (21.2%). The most diverse polymorphism was 448G>T with a frequency of 49.9% for heterozygous. The six polymorphisms were found in genetic equilibrium and a genetic structure of populations was not evidenced (F_ST_= 0.0038).

**Conclusion:**

The population studied does not present a genetic sub-structure and the polymorphisms analyzed were found in genetic equilibrium. This indicates that the population mixes randomly and there are no sub-groups capable of affecting the results of the association studies.

## Abstract (es)

**Introducción:**

A pesar que cerca del 40% de la variabilidad en la presión arterial es explicada por factores genéticos, la identificación de genes asociados a la hipertensión arterial esencial es difícil en poblaciones constituidas por individuos con antecedentes genéticos diferentes; en esta circunstancia se debe determinar si la población está sub-estructurada porque esto puede sesgar los estudios de asociación con esta enfermedad.

**Objetivo:**

Determinar la estructura genética de la población de Bucaramanga a partir de polimorfismos genéticos asociados con la regulación de la presión arterial: 448G>T, 679C>T y 1711C>T del gen de la quinasa 4 del receptor dopaminérgico acoplado a proteína G y Glu298Asp, -786T>C y el VNTR del intrón 4 del gen de la sintasa de óxido nítrico endotelial.

**Métodos:**

Se estudió una muestra de 552 individuos no relacionados mediante análisis de polimorfismos de longitud de fragmentos de restricción. Se calcularon las frecuencias alélicas, haplotípicas y genotípicas, se determinó el equilibrio de Hardy-Weinberg y se realizó un análisis molecular de varianza para determinar la estructura genética.

**Resultados:**

Se identificaron 38 haplotipos siendo GCCTG4b el más frecuente (21.2%). El polimorfismo más diverso fue el 448G>T con una frecuencia de heterocigotos del 49.9%. Los seis polimorfismos se encontraron en equilibrio genético y no se evidenció estructura genética poblacional (F_ST_ = 0.0038).

**Conclusión:**

La población estudiada no presenta subestructura genética y los polimorfismos analizados se encontraron en equilibrio genético, lo que indica que la población se mezcla aleatoriamente y no existen subgrupos que puedan afectar los resultados de estudios de asociación.

## Introduction

Blood pressure levels tend to aggregate in families due in part to shared genetic predispositions. In fact, about 40% of the variability in blood pressure is explained by genetic factors and the risk of developing it after age 50 doubles for each first-degree relative with a history of hypertension^1^. Blood pressure is regulated by multiple mechanisms involving several non-allelic genes with small additive effects. Although the specific mechanism altered cannot be identified in about 90% of cases, the individual genetic variants (alleles) or combinations of alleles (haplotypes) involved in the regulation of blood pressure are genetic factors with more likelihood of increasing the risk of developing hypertension.

Genetic variants or polymorphisms associated with the regulation of urinary excretion of sodium and vasomotor regulation are potential risk factors for the development of hypertension. Among the former are the polymorphisms 448g>T or R65L or rs2960306, 679C>T or A142V or rs1024323 and 1711C>T or A486V or rs1801058 of the gene GRK4 that encodes the kinase 4 of receptors coupled to the G protein, specifically D1 and D2 dopamine receptors, which mediate the natriuretic effect of catecholamine in the proximal convoluted tubule of the nefron^2^. Among the latter are the highlighted polymorphisms of 894G>T or Glu298Asp or rs1799983, the - 786T>C or rs2070744 and Intron 4 of the gene eNOS that encodes the endothelial nitric oxide synthase^3^. Nitric oxide (NO) induces vasodilation and reduction of blood pressure by inhibiting the growth and contraction of the smooth muscle arterial wall^4^. The relationship of these polymorphisms with the risk of developing hypertension is still uncertain^5^.

One of the obstacles in identifying genetic variants associated with hypertension is the comparison of cases and controls that come from populations with different genetic backgrounds. This problem is known as genetic structure and generates a selection bias due to cases and controls having a different distribution of alleles of the polymorphisms associated with the disease of interest^6^ ^-^ ^8^. Consequently, various methods have been proposed to identify and control the population structure in genetic association studies^9^.

Taking into account that the national and local context are minimal, the studies involving the aforementioned polymorphisms, likewise the deficiency of research to analyze participation in the genetic structure in our population led to a population genetic study being designed in Bucaramanga. It starts with genotyping of the polymorphisms 448G>T, 679C>T and 1711C>T of the gene GRK4 and Glu298Asp,-786T>C and intron 4 of the eNOS gene in order to establish the degree of genetic structure for this population. It is expected that these results will give support to subsequent association studies of these polymorphisms with HAE in the population of Santander and thus avoid potential bias regarding the possibility of finding associations that could be false.

## Methods and Materials

### Study Population

Five hundred fifty-two (552) participants were selected for the INEFAC (Incidence of Cardiovascular Disease and Risk Factors in Colombian) project, a cohort study with random sampling of residents of Bucaramanga, Colombia from the lower socio-economic strata of 2 and 3. The sample included 372 women and 180 men between the ages of 16 and 69 years with an average of 35.1 years and normotensive (systolic blood pressure <120 mmHg and diastolic blood pressure <80 mmHg). This study was approved by the ethics committee of the School of Health from the Universidad Industrial de Santander and all participants gave their written informed consent.

The sample calculations were performed taking into account the frequency of the minor allele prevalence for the six polymorphisms studied in different populations^10^ ^-^ ^13^. Further, parameters were established with a confidence level of 95%, power of 86%, relative risk expected (RRE) of 2.0 and relative case control: 1:1.

### DNA extraction and bioinformatic methods

From all individuals a sample was taken of peripheral blood with EDTA anticoagulant and from it DNA was extracted by the phenol-chloroform method^14^. Polymorphisms of genes GRK4 and eNOS were amplified by means of polymerase chain reaction (PCR) and were identified by means of enzyme restriction through identification of the size of the resulting fragments (RFLP's)^15^. The genomic sequences of the genes GRK4and eNOS and polymorphisms of interest were verified on the database of the National Center for Biotechnology Information (NCBI) of the United States (http://www.ncbi.nlm.nih.gov) and (http://www.ncbi.nlm.nih.gov/projects/SNP). The location of the places where the enzyme cuts were made were verified with the Restriction Mapper software (http://www.restrictionmapper.org/).

### PCR amplification and genotyping of SNPs

The sequences of the primers for amplifying each polymorphism are noted in Table 1. The discordant PCR-RFLP technique was used to detect the polymorphism 679C>T and 1711C>T, in which a base was changed in one of the primers so that a fragment was generated which differed from a base with respect to the DNA template. Consequently, the amplified product resulting from the ancestral allele acquired a restriction site which is not present in the mutating allele^16^.

**Table 1:**
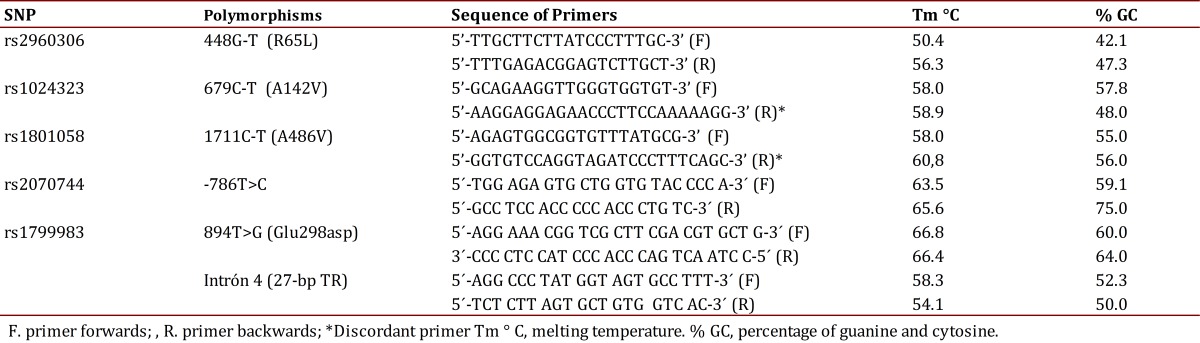
Specifications of the primers

The PCR reaction for the polymorphisms of the gene GRK4 contained 1X buffer, 3.5 mM MgCl_2_, 0.5 µM of each primer, 0.8 µM of deoxyribonucleotide triphosphate (dNTP), 1 U of enzyme Taq DNA polymerase (Promega®) and 3.9 ng of DNA in a final volume of 10 µL. The amplification protocol included an initial step of 94° C for 5 min, followed by 38 cycles as follows: denaturation at 95° C for 15 seconds, annealing at 60° C for 15 seconds and extension at 72° C for 30 seconds and a final step at 72° C for 7 min. In each PCR reaction amplification used negative controls and positives were used for the wild-type and mutant alleles. The PCR products were verified by electrophoresis in a 1% agarose gel including a molecular weight marker of 50 to 500 bp, to check the size of the amplified sample.

The PCR products were subjected to restriction using the enzymes reported in Table 2, under the following protocol: 1X buffer, 0.1 µg/µL of acetylated BSA, 1 U of the corresponding restriction enzyme and 0.2 ng of the amplified DNA, at a final volume of 10 µL; the reaction was carried out by incubation for 10 hours at 37° C^13^.

**Table 2:**
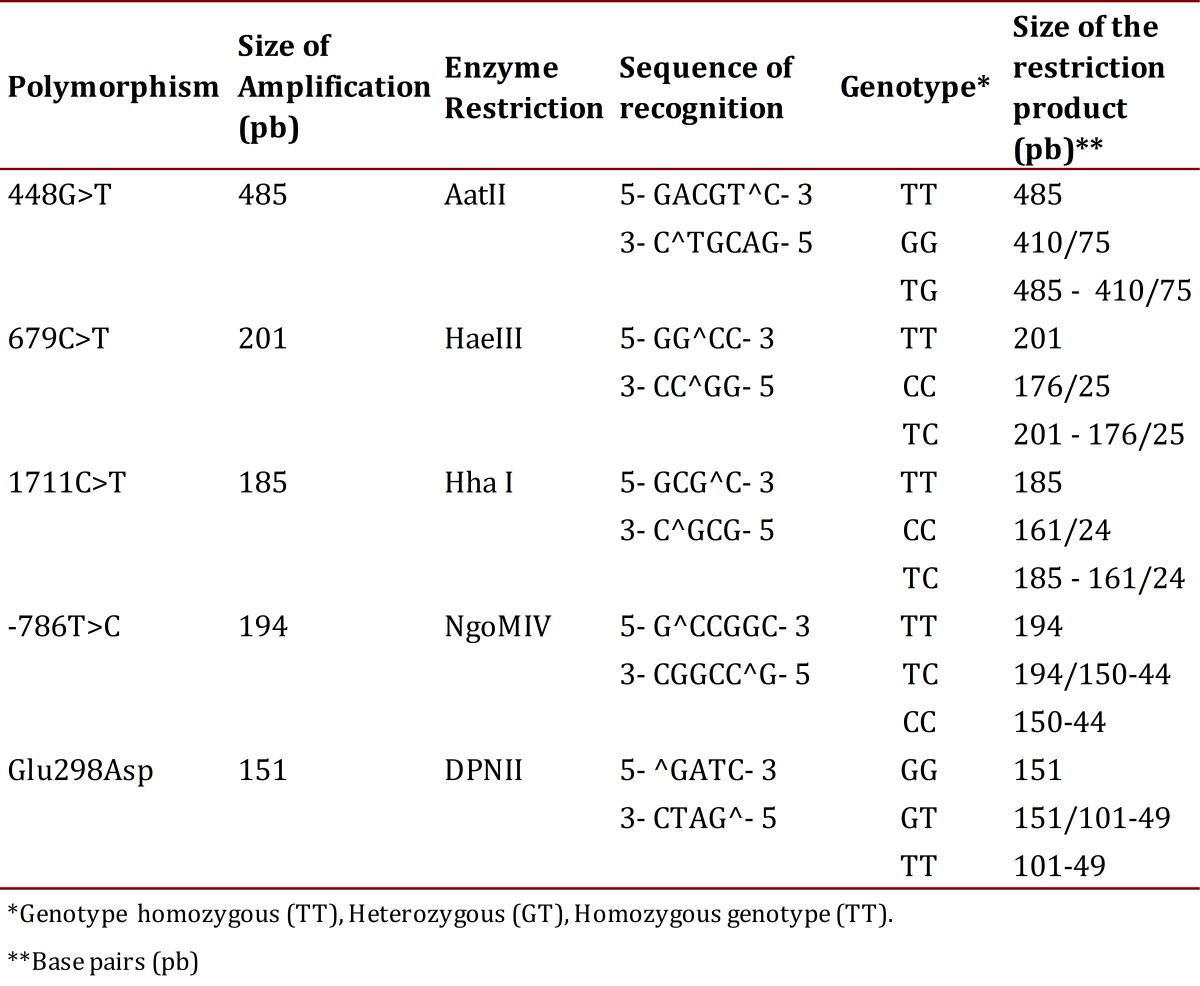
Restriction enzymes and the sizes of the restriction products

The set-up of the Glu298Asp polymorphism PCR contained 1X Buffer-GoTaq (r) - Green Master Mix 0.4 µM of each primer and 2.4 ng of DNA, for a final volume of 25 µL. The amplification protocol consisted of an initial step of 95° C for 2 min, followed by 35 cycles as follows: denaturation at 95° C for 45 seconds, annealing at 63° C for 45 seconds and extension at 72° C for 45 seconds and a final step at 72° C for 5 min. The PCR set-up for polymorphism -786T>C containing 1X Buffer, 2.5 mM MgCl_2_ , 0.4 µM of each primer, 0.8 µM dNTPs, 1 U of enzyme Taq DNA polymerase (promega®) and 2.4 ng of DNA in a final volume of 25 µL. The amplification protocol included an initial step of 94° C for 4 min, followed by 35 cycles as follows: denaturation at 94° C for 30 seconds, annealing at 63° C for 30 seconds and extension at 72° C for 1 min and a final step at 72° C for 5 min. The amplification of these polymorphisms was verified by electrophoresis in a 1% agarose gel. The PCR products were subjected to enzyme restriction using the following protocol: 1X buffer, 0.1 µg/µL of acetylated BSA, 1 U of the corresponding restriction enzyme (Table 2) and 0.5 ng of DNA in a final volume of 15 µL; the reaction was conducted for incubation during 14 hours at 37° C.

The products of the enzymatic digestion of the two SNPs studied were separated by means of electrophoresis in 3% agarose gels and for visualization of the bands ethidium bromide was added. The gels were run in an electrophoresis chamber Power Pac 300 (BioRad®) in a 1X TBE buffer and 60 volts were applied for 80 min. Recognition sites for enzymes, the sizes of the expected fragments and the assigned genotypes are shown in Table 2.

The set-up of the PCR for intron 4 was performed using the same protocol as the polymorphism-786T>C. The sizes of the fragments of this polymorphism, amplification products were visualized by means of electrophoresis in a 3% agarose gel with a tension of 60 volts for 80 min. In Table 3 displays the size of the amplification products of this intron^17^. The analyses of all samples were performed in a blind manner to avoid bias and 10% of the samples were processed in duplicate with no discrepancy being evident.

**Table 3:**
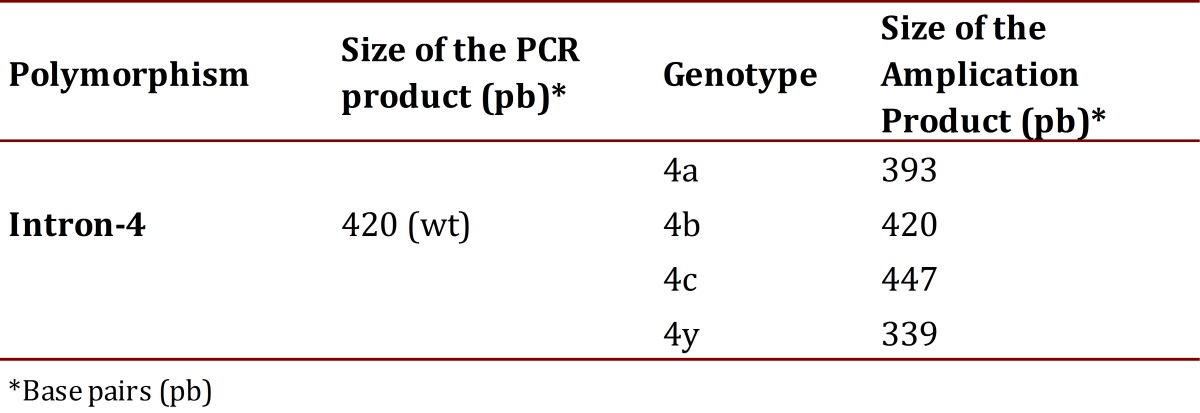
Product size for Intron 4

### Sequencing

Once the PCR was standardized, six samples were selected of each polymorphism to be sequenced with the Big Dye terminator kit (Applied Biosystems®) and the sequences obtained were aligned with the Clustal W software (http://www.ebi.ac.uk selected/Tools/msa/clustalw2/) to confirm correspondence with the expected fragments.

### Statistical analysis

With the results obtained from reading the electrophoresis, two databases were compiled using Microsoft Excel® 2007 validated by the Data Compare software Epi Info, version 3.5.1^18^ and the genotype was established for each polymorphism typified in the samples. For each of the studied polymorphisms these data were used to calculate the genotype frequencies with the GenAlex 6.3® program^19^; allele frequencies, haplotype frequencies, the Hardy-Weinberg Equilibrium test (HWE) and the analysis of molecular variance (AMOVA) to determine the presence of the genetic structure of the population. All of this was carried out using the Arlequin v 3.5 program^20^; finally, the genetic structure of the population was verified by using the program, Structure v 2.3^21^.

## Results

**Figure 1:**
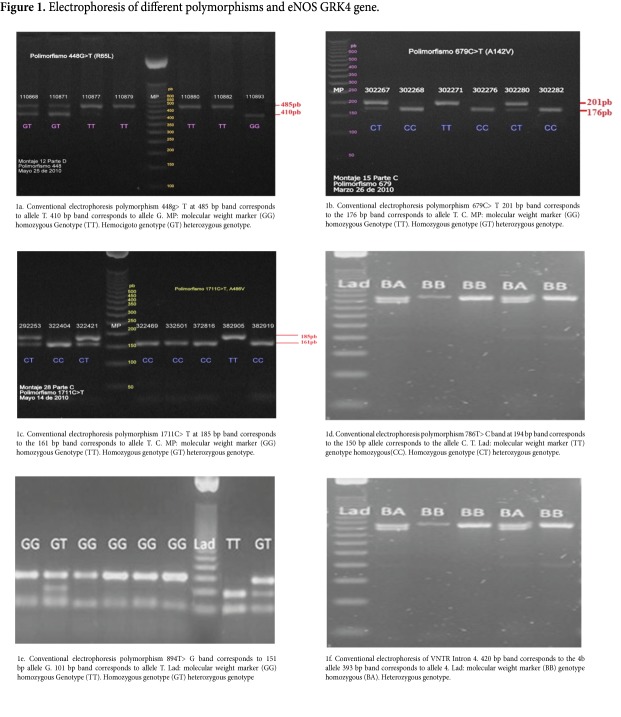
Electrophoresis of different polymorphisms and eNOS GRK4 gene

The RFLP typing of the different analyzed polymorphisms allowed the detection of all possible genotypes for each one of them in the study population (Fig. 1). From the observed genotypes, genotype and allele frequencies were calculated for each of the polymorphisms of the genes GRK4 and eNOS. Also calculated were the haplotype frequencies for the combined six polymorphisms. It was established that all polymorphisms were in HWE as the *p* values found ​​were greater than 0.05 (Table 4).

**Table 4:**
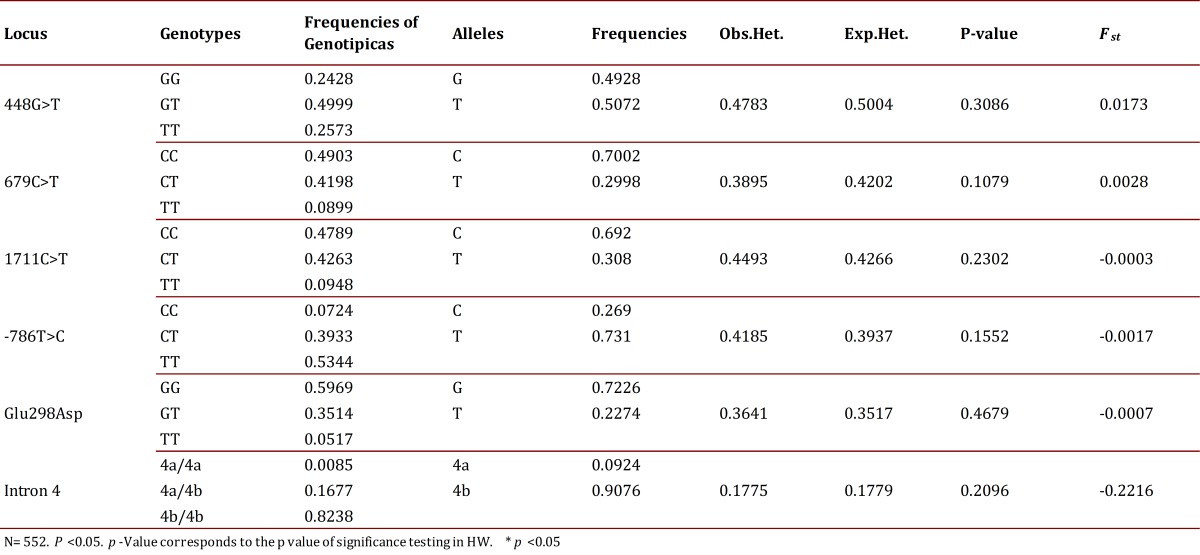
Genotype frequencies, alleles and HWE

According to the hierarchical AMOVA, no genetic structure (F_ST_= 0.0038) was evident in the analyzed sample. Additionally, it was not observed that the F_ST_ values ​​obtained for each polymorphism studied (Table 4) significantly contributed to the differentiation of the population^22^.

**Figure 2:**
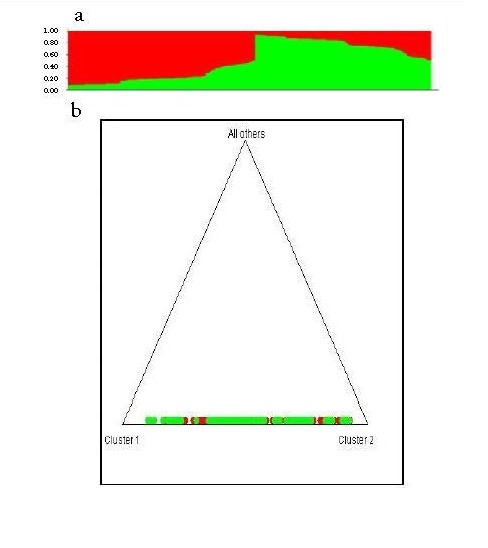
Structure analysis in 552 samples of individuals from the population of Bucaramanga from typing six polymorphisms associated with hypertension. The 2nd. Bar plot of the possible mixing of the 552 individuals; 2b. Triangle plot showing individuals distributed in a single genetic unit.

Evidence for the lack of structure was confirmed by the analysis performed with the Structure v. 2.3 software to evaluate K=2 (possible ancestors) and with 10,000 replicates assuming a mixed model^21^. The results showed that very few individuals who could possibly belong to another population group, they also showed the absence of genetic structure in the population sample analyzed (Figs. 2A and 2B).

In the estimation of haplotype frequencies for the six polymorphisms studied, it was found that the most frequent was GCCTG4b (21.2%) from a total of 38 haplotypes detected in the 1,104 chromosomes analyzed (Table 5).

**Table 5:**
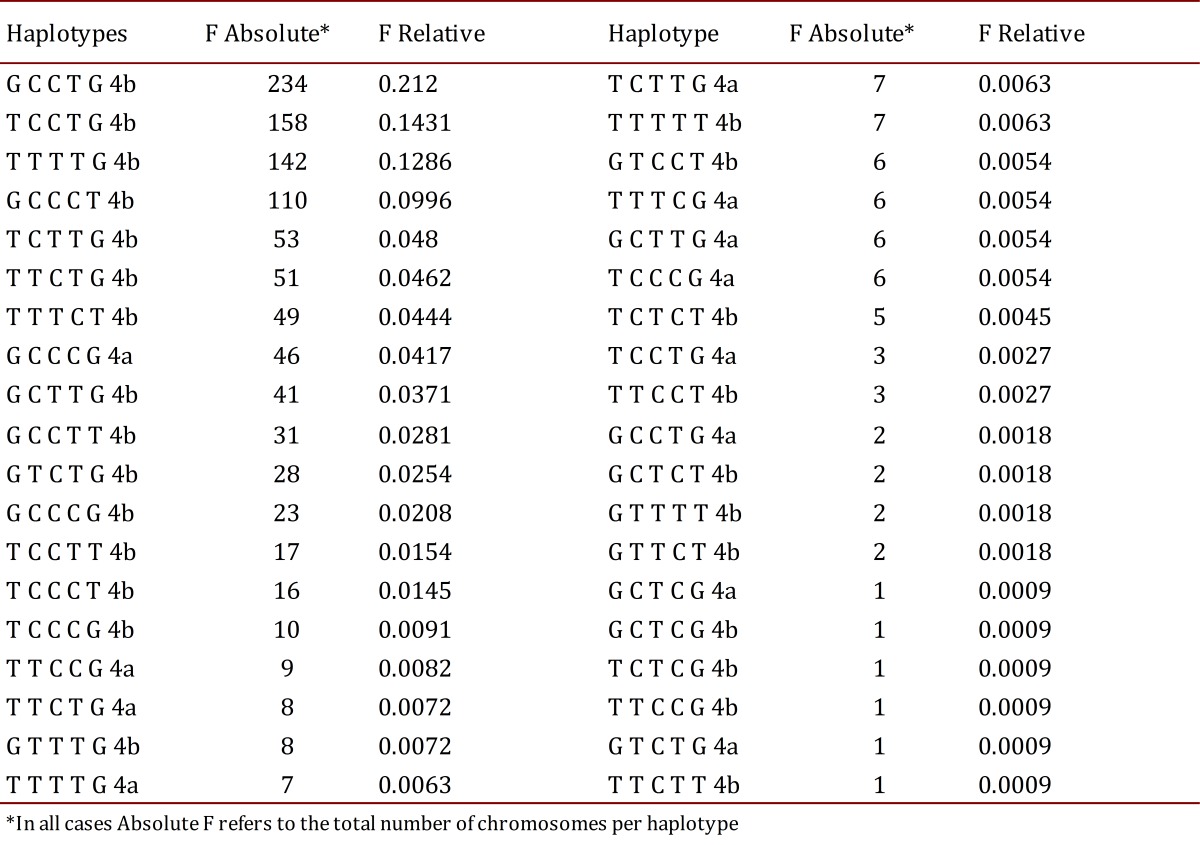
Frequency of haplotypes of polymorphisms of the kinase 4 (GRK4) and ENOS gene.

## Discussion

According to the values ​​obtained for the allele frequencies in the study population, the most common allele for each polymorphism of the GRK4 gene was: 448g>T allele T (50.7%), 679C>T allele C (70%), 1711C>T C allele (69%); in a previous study conducted on a Hispanic population in Southern California, the results coincide for the polymorphisms 679C>T and 1711C>T and differ for polymorphism 448g>T^23^.

The most frequent allele in each polymorphism of the eNOS gene was: -786T>C allele T (76%), Glu298Asp allele G (72%) and intron 4 allele 4b (90%). These results are consistent with those found in a prior study performed on the Bucaramangan population^15^.

In the present study 38 haplotype combinations were found, the most frequent being GCCTG4b with 21.2%. No publications were found on populations where haplotype frequencies were reported for these six polymorphisms in Hispanic or Colombian populations, making this study the first report thereof.

All studied polymorphisms were found in the Hardy-Weinberg Equilibrium indicating that the population is composed of individuals that are mixed randomly. This finding agrees with that reported in previous studies on a Bucaramangan population for the gene eNOS^15^ and in an Hispanic population of Southern California for the gene GRK4^23^ where no data are reported for the Colombian population.

Additionally, no population structure was found in the analyzed sample, which coincides with that reported in a previous study conducted on the population in the city of Bucaramanga from the analysis of other genetic polymorphic markers^24^.

## Conclusions

The results found confirm that the study population was found in HWE for all systems studied and do not present population substructure, which allows for further association study of these polymorphisms with essential hypertension since it is clear that the associations between candidate genes to develop multi-factorial diseases must be interpreted within the context of the genetic structure of the population being studied.
